# Immune dysregulation linking pre-pregnancy obesity to gestational diabetes mellitus

**DOI:** 10.3389/fimmu.2026.1940789

**Published:** 2026-09-07

**Authors:** Hanna Margrethe Storheil Skarstad, Md Abu Jafar Sujan, Trine Moholdt, Michael Z. Zulu

**Affiliations:** 1 Department of Circulation and Medical Imaging, Norwegian University of Science and Technology, Trondheim, Norway; 2 Department of Women’s Health, St. Olav’s Hospital, Trondheim University Hospital, Trondheim, Norway; 3 Department of Medicine, St. Olav’s Hopsital, Trondheim, Norway; 4 Department of Virology, School of Pathology, Faculty of Health Sciences, University of the Witwatersrand, Johannesburg, South Africa; 5 Institute for Infectious Disease and Molecular Medicine, Faculty of Health Sciences, University of Cape Town, Cape Town, South Africa

**Keywords:** gestational diabetes mellitus, immune dysregulation, obesity, pregnancy, pre-pregnancy

## Abstract

Gestational diabetes mellitus (GDM) is a prevalent metabolic complication of pregnancy with rising global incidence. Its increasing prevalence parallels the rising rates of pre-pregnancy obesity worldwide. While physiological insulin resistance is a normal feature of pregnancy, excess maternal adiposity exacerbates this process and significantly increases the risk of GDM. Emerging evidence highlights immune dysregulation as a plausible mechanistic link between pre-pregnancy obesity and impaired metabolic adaptation during pregnancy. Obesity is characterised by chronic, low-grade inflammation, driven by adipose tissue expansion, immune cell infiltration, and dysregulated cytokine and adipokine production. These changes may collectively impair insulin signalling, disrupt placental function, and alter maternal–foetal immune tolerance. In this review, we synthesise current understanding of the immunological pathways connecting pre-pregnancy obesity to GDM, including alterations in innate and adaptive immune responses, soluble inflammatory mediators, and placental immune dynamics. By integrating metabolic and immunological perspectives, this narrative mini-review highlights potential mechanisms underpinning obesity-associated GDM and highlights critical gaps in knowledge relevant to early risk stratification and targeted prevention strategies.

## Introduction

Gestational diabetes mellitus (GDM), defined as glucose intolerance first recognised during pregnancy, represents one of the most common metabolic complications of pregnancy and a growing global public health concern (1). Its prevalence has risen substantially alongside increasing maternal age, sedentary lifestyles, and, most notably, obesity rates among women of reproductive age (2). Current estimates suggest that GDM affects approximately 5–20% of pregnancies worldwide, with considerable geographic variation depending on diagnostic criteria and population risk profiles (3). Concurrently, pre-pregnancy obesity, a major risk factor for GDM (4), has reached epidemic proportions, with a significant and increasing proportion of women entering pregnancy with excess adiposity. GDM, particularly when occurring in the context of maternal obesity, has profound implications for both maternal and offspring health, including foetal overgrowth, obstetric complications, and type 2 diabetes mellitus and cardiovascular diseases in later life (5–9). While insulin resistance is a normal physiological adaptation of pregnancy, obesity amplifies this state through mechanisms that extend beyond altered glucose and lipid metabolism (10). Increasing evidence identifies immune dysregulation as a possible central pathophysiological link between obesity and GDM (11, 12). Obesity-associated chronic, low-grade inflammation is driven by adipose tissue expansion, immune cell infiltration, altered cytokine and adipokine secretion (13). This inflammatory state impairs insulin signalling, disrupts placental function, and interferes with maternal–foetal immune tolerance (13). Pre-existing inflammatory and immune alterations associated with obesity may therefore promote insulin resistance and β-cell dysfunction, increasing susceptibility to GDM in women with obesity (14).

In this review, we synthesise current evidence on the immunological mechanisms linking pre-pregnancy obesity to GDM by examining the immune adaptations that characterise normal pregnancy and how these processes are disrupted by maternal obesity. We further evaluate the contribution of cellular immune pathways, soluble mediators, and placental alterations to the impaired metabolic adaptation associated with maternal obesity. Relevant literature published over the past 15 years was identified through a non-systematic search of databases, including PubMed and Scopus, using search terms related to “*gestational diabetes*”, “*placenta*”, “*maternal obesity*”, and “*immune dysregulation*”. Priority was given to original research articles and relevant review papers.

This review adopts an immunometabolic framework in which pre-pregnancy obesity is considered the initiating event that establishes a chronic state of low-grade immune activation before conception. We propose that a pre-existing inflammatory milieu impairs normal immunological and metabolic adaptations required during pregnancy. Within this framework, obesity-induced immune dysregulation is proposed as a potential pathway contributing to GDM susceptibility. Placental inflammation and further immune activation after the onset of hyperglycaemia may represent amplifying mechanisms that worsen disease progression and contribute to adverse maternal and foetal outcomes. Accordingly, this review follows a temporal progression from pre-pregnancy obesity and immune dysregulation, to impaired maternal immunometabolic adaptation, GDM development, and downstream placental and offspring effects. By integrating immunological and metabolic perspectives, we highlight possible mechanistic links, identify knowledge gaps, and potential targets for early risk stratification and prevention of obesity-associated GDM.

## Immunological adaptations in normal pregnancy

Pregnancy represents a unique immunological state requiring a balance between maternal tolerance towards the semi-allogeneic foetus and effective pathogenic defence (15, 16). Successful gestation depends on coordinated adaptations in both systemic immunity and the uterine microenvironment to support implantation, placental development, and foetal growth (17). Disruption of the tightly regulated immunological balance is associated with adverse outcomes, including GDM, pre-eclampsia and preterm birth (16, 18). Central to these adaptations is the maternal-foetal interface, a specialised immunological microenvironment comprising maternal decidua and foetal-derived placental tissue (19). The maternal-foetal interface undergoes dynamic structural and immunological changes throughout gestation, maintaining tolerance while permitting the controlled immune activation required for tissue remodelling and parturition (19, 20). Longitudinal studies confirm that maternal immune responses follow trimester-specific trajectories, characterised by pro-inflammatory phases in early and late pregnancy and an anti-inflammatory state during mid-gestation (21).

During early pregnancy, decidualisation and trophoblast invasion facilitate placental formation and vascular remodelling, processes that are closely regulated by local immune cells (18, 19). Rather than representing immune ignorance, maternal-foetal tolerance is now understood as an active and regulated process (17). Pregnancy induces foetal antigen-specific immune responses that are tightly controlled by regulatory mechanisms (22, 23). Regulatory T cells (Tregs) play a central role by suppressing excessive effector T-cell and natural killer (NK) cell activity at the maternal-foetal interface (24–26).

Immune cell populations at the maternal-foetal interface are highly specialised and functionally adapted (17, 18). Decidual NK (dNK) cells constitute a major immune subset in early pregnancy and play key roles in trophoblast invasion and spiral artery remodelling (27). Decidual macrophages contribute to tissue homeostasis, clearance of apoptotic cells, and maintenance of immune tolerance, while Treg cells suppress inflammatory responses and promote tolerance to foetal antigens (28). Together, these cells create a controlled immunological environment that supports placental development and foetal growth.

Decidual T cells comprise functionally distinct subsets essential to the maintenance of pregnancy (29, 30). Regulatory CD4+ T cells (CD25^hi^FOXP3+) are enriched in the decidua relative to peripheral blood and are associated with healthy pregnancy outcomes (20, 22, 31), thus playing a central role in establishing and maintaining immune tolerance at the maternal-foetal interface. Additionally, these cells suppress excessive inflammation and modulate local immune cell activity, thereby limiting immune-mediated damage to foetal tissue and helping to maintain tolerance to foetal alloantigens (32).

Decidual macrophages represent one of the most abundant antigen-presenting cells (APCs) in the human decidua, comprising approximately 20-30% of decidual leukocytes during the first trimester (33). Here, they play a central role in promoting tolerance to the semi-allogeneic foetus and maintaining the homeostatic environment required for normal foetal development (34). Dendritic cells (DCs) represent another APC population whose role remains comparatively poorly defined. While murine models suggest that uterine DCs exhibit high expression of antigen-presenting and T-cell-stimulating markers, human decidual DCs demonstrate functional capacity to activate T cells ex vivo suggesting a role in balancing immune tolerance and activation during pregnancy (35, 36). However, the precise roles of decidual macrophages and T cells throughout gestation remain incompletely understood. Although the contributions of individual immune cell populations require further investigation, the coordinated adaptation of the maternal immune system is essential for normal placental development and maintenance of metabolic homeostasis. This physiological immunological state provides an important baseline for understanding how pre-pregnancy obesity disrupts maternal immune regulation and contributes to adverse metabolic outcomes during pregnancy.

## Obesity-induced immune dysregulation before and during pregnancy

Before conception, individuals with obesity often exhibit chronic low-grade inflammation characterised by sustained immune activation and altered adipokine signalling, reflected by elevated circulating inflammatory markers and mediators, including C-reactive protein, interleukins (IL), interferons, and tumour necrosis factor (TNF) (37–40). Adipose tissue functions as an endocrine and immunological organ, secreting cytokines, chemokines and adipokines (41, 42). Excess energy availability promotes adipose tissue expansion through adipocyte hypertrophy and hyperplasia, leading to structural and metabolic alterations within adipose tissue (43, 44). These changes induce cellular stress, including lipotoxicity, oxidative stress, and hypoxia, with consequent release of free fatty acids and inflammatory mediators that amplify local and systemic inflammation (44, 45).

Adipocytes are the principal functional cells within adipose tissue and secrete peptide hormones collectively referred to as adipokines, thereby playing a central role in linking metabolism and immune regulation (41). Leptin concentrations increase with adiposity and promotes immune cell activation and pro-inflammatory cytokine production (46, 47). Adiponectin exerts anti-inflammatory and insulin-sensitising effects, and is reduced in obesity and metabolic disease (48, 49). This shift in adipokine balance contributes to a pro-inflammatory state that favours insulin resistance. Concurrently, macrophages in the stromal vascular fraction of adipose tissue act as central mediators of obesity-associated inflammation, and increased infiltration of pro-inflammatory macrophages further amplifies adipose tissue inflammation through cytokine and chemokine secretion (44, 50–52).

In reproductive-aged females, these immunometabolic disturbances carry significant implications for pregnancy (**Table 1**). Because successful gestation requires tightly regulated immune adaptation, pre-existing obesity-associated inflammation may disrupt this balance by increasing pro-inflammatory cytokines, adipokines, and immune cell activation, including increased NK cell activation and TNF-α production, thereby heightening the risk of adverse pregnancy outcomes such as GDM and pre-eclampsia (64). Consequently, individuals with obesity may enter pregnancy with a pre-existing inflammatory milieu that may compromise the normal immunometabolic adaptations required during gestation.

**Table 1:** Maternal obesity and its impact on maternal, placental, and offspring health.

Stage	Changes associated with maternal obesity	Impact on maternal, placental, and offspring health	References
Pre-pregnancy obesity	↑ C-reactive protein (CRP)	Reflects chronic low-grade systemic inflammation associated with insulin resistance, endothelial dysfunction, and increased risk of pregnancy complications.	(10)
	↑ Pro-inflammatory cytokines (IL-6, IL-1β, TNF-α)	Promote chronic inflammation, immune activation, and metabolic dysfunction before conception.	(53, 54)
	↑ Leptin	Contributes to leptin resistance, impaired metabolic regulation, and enhanced inflammatory responses.	(55, 56)
	↓ Adiponectin	Reduces insulin sensitivity and anti-inflammatory signalling, increasing susceptibility to gestational diabetes mellitus (GDM).	(57, 58)
Pregnancy complicated by obesity	↑ M1-like adipose tissue macrophages	Promotes chronic adipose tissue inflammation and secretion of pro-inflammatory mediators.	(59, 60)
	↑ Maternal IL-6, TNF-α and other inflammatory mediators	Exaggerates systemic inflammation and contributes to insulin resistance and endothelial dysfunction during pregnancy.	(54, 61)
	↑ Placental macrophage activation (Hofbauer cells)	Enhances placental inflammatory signalling and alters placental immune homeostasis.	(53)
	↑ Toll-like receptor (TLR2/TLR4) signalling	Activates innate immune pathways leading to increased NF-κB activation and cytokine production.	(53, 54)
	Altered maternal T-cell responses	Disrupts maternal immune tolerance and contributes to placental inflammation.	(54)
	Placental dysfunction	Increased inflammatory cytokines, oxidative stress, impaired angiogenesis, altered nutrient transport, and placental vascular dysfunction.	(61, 62)
	Offspring consequences	Increased risk of foetal overgrowth, metabolic syndrome, obesity, insulin resistance, immune dysregulation, and neurodevelopmental disorders later in life.	(63)

CRP, C-reactive protein; GDM, gestational diabetes mellitus; IL, interleukin; NF-κB, nuclear factor kappa B; TNF-α, tumour necrosis factor-alpha; TLR, Toll-like receptor. "↓" means "decrease" and "↑" means "increase".

## Immune mediators linking obesity to GDM

Pregnancy is characterised by progressive physiological insulin resistance which increases maternal glucose availability for foetal nutrition. This process is normally compensated by increased pancreatic β-cell insulin secretion. Individuals with pre-pregnancy obesity, however, already exhibit chronic low-grade inflammation characterised by altered adipokine secretion, macrophage activation, and elevated pro-inflammatory cytokines. These pre-existing immune abnormalities reduce metabolic flexibility and impair the maternal adaptations required to maintain glucose homeostasis, thereby increasing susceptibility to GDM (11, 65).

When maternal metabolic adaptive capacity is exceeded, pro-inflammatory cytokines, adipokines, and innate immune signalling pathways act in a coordinated manner to disrupt insulin receptor signalling and impair β-cell compensation. These processes can amplify metabolic dysfunction across adipose tissue, the liver, skeletal muscle and placenta.

Tumor Necrosis Factor alpha (TNF-α), Interleukin-6 (IL-6), and Interleukin-1 beta (IL-1β) are frequently elevated in GDM and may contribute to obesity-associated insulin resistance (12, 66). TNF-α impairs insulin signalling and promotes lipolysis, increasing circulating free fatty acids that further exacerbate insulin resistance (67, 68). In pregnancy, maternal TNF-α concentrations correlate with indices of insulin resistance and are elevated in GDM (69). IL-6 may impair hepatic insulin sensitivity and adversely effect pancreatic β-cell function (70, 71). Placental overproduction of IL-6 during pregnancy may exacerbate physiological insulin resistance as it is associated with increased GDM risk (70, 71). IL-1β, activated through the NOD-like receptor pyrin 3 (NLRP3) inflammasome under obesity-induced lipotoxicity and oxidative stress, impairs peripheral insulin action and decreases β-cell insulin secretion, potentially contributing to failure of the adaptive β-cell response in GDM (72, 73).

Adipokines further modulate immune–metabolic interactions. Increased leptin and reduced adiponectin levels promote pro-inflammatory signalling and insulin resistance, while altered resistin expression may contribute to hepatic glucose dysregulation (65). Collectively, these immune mediators form an interconnected network that, in the context of pre-pregnancy obesity, could disrupt insulin signalling and limit β-cell compensation, thereby increasing GDM susceptibility.

## Immune pathways in obesity-associated GDM

**Figure 1:**
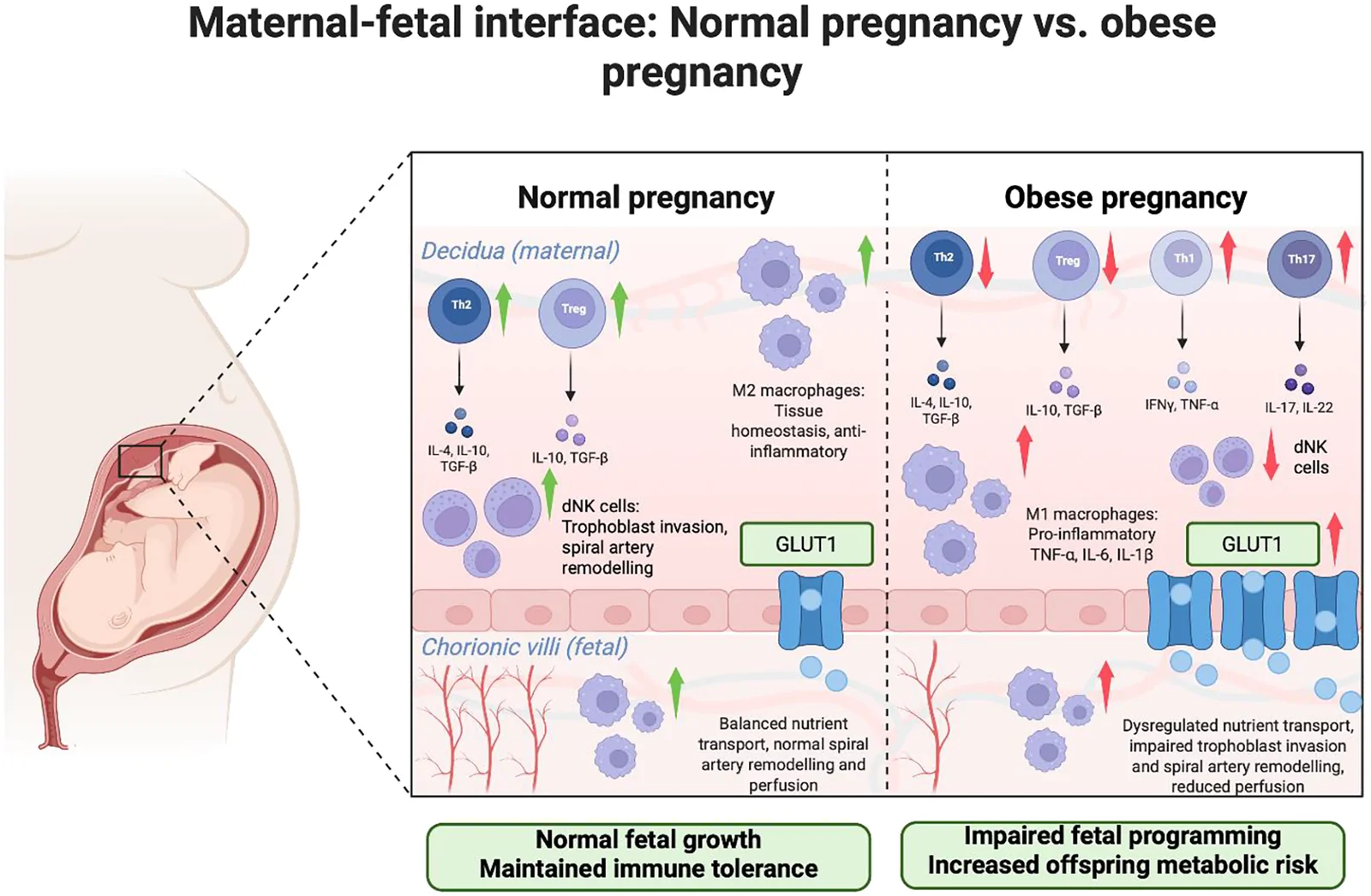
Maternal–foetal interface in normal pregnancy versus obesity-complicated pregnancy. Schematic representation of the immunological and metabolic adaptations at the maternal–foetal interface during normal pregnancy (*left*) and maternal obesity (*right*). In normal pregnancy, the decidual microenvironment is characterised by enrichment of T helper 2 (Th2) cells, regulatory T (Treg) cells, decidual natural killer (dNK) cells, and alternatively activated M2 macrophages, which promote immune tolerance, trophoblast invasion, spiral artery remodelling, and tissue homeostasis through the production of anti-inflammatory cytokines, including IL-4, IL-10, and TGF-β. These adaptations support balanced nutrient transport, adequate placental perfusion, and normal foetal growth. In contrast, maternal obesity is associated with a shift towards a pro-inflammatory milieu, characterised by increased Th1 and Th17 responses, reduced Treg and dNK cell frequencies, and accumulation of classically activated M1 macrophages producing TNF-α, IL-6, IL-1β, IFN-γ, IL-17, and IL-22. Obesity is also associated with altered placental GLUT1 expression and nutrient transport, impaired trophoblast invasion and spiral artery remodelling, and reduced placental perfusion. Collectively, these changes disrupt maternal–foetal immune tolerance and placental function, contributing to abnormal foetal programming and an increased risk of metabolic disease in the offspring. Green arrows indicate increased abundance or activity, while red arrows indicate decreased abundance or activity relative to normal pregnancy. dNK, decidual natural killer; GLUT1, glucose transporter 1; IFN-γ, interferon-γ; IL, interleukin; TGF-β, transforming growth factor-β; TNF-α, tumour necrosis factor-α. **Figure 1** represents a conceptual scheme based on original data synthesised in this review. Created in Biorender.com.

Following the onset of impaired glucose homeostasis, innate and adaptive immune pathways further amplify inflammation and metabolic dysfunction. Macrophages are central mediators of innate immune dysregulation in obesity and GDM. Metabolic stressors such as hyperglycaemia, elevated free fatty acids and oxidative stress drive pro-inflammatory macrophage activation via TLR signalling (74–78). These pathways promote inflammatory cytokine production and activation of NF-κB and JNK signalling, thereby contributing to insulin resistance. In individuals with obesity, increased macrophage infiltration in adipose tissue and placenta is accompanied by elevated circulating levels of TNF-α, IL-6, and IL-1β (12, 53). Placental studies in GDM also demonstrate increased macrophage abundance and pro-inflammatory activity, potentially impairing vascular development and nutrient transport (53). Circulating mediators such as macrophage migration inhibitory factor (MIF) may additionally amplify inflammation and macrophage recruitment (79). Placental macrophages, known as Hofbauer cells (HBCs) display immunoregulatory functions and support placental vascular development in healthy pregnancies (80), but may adopt a more pro-inflammatory phenotype in GDM (81). However, their precise role in GDM remains incompletely characterised (82) (**Figure 1**).

Innate immune activation is further amplified through TLR signalling and inflammasome activation. Increased expression of TLRs and activation of the NLRP3 inflammasome promote caspase-1–mediated maturation of IL-1β and IL-18, contributing to placental inflammation and systemic metabolic dysfunction (76, 83). In addition, complement activation has been associated with systemic inflammation and metabolic dysfunction in GDM, suggesting a broader role for innate immune pathways in disease pathogenesis (84, 85).

Adaptive immunity is similarly dysregulated in obesity-associated GDM, characterised by an imbalance between pro-inflammatory effector T cells and Treg cells. Expanded Th1 and Th17 responses elevate IFN-γ, IL-17, and TNF-α, while Treg-cell abundance and function are reduced in GDM (86–92). A 2023 meta-analysis by Arain and colleagues further demonstrated a significant reduction in circulating Treg cells in women with GDM, supporting their potential utility as biomarkers for risk stratification (93).

Chronic exposure to pro-inflammatory cytokines in obesity may impair Treg function and reduce immunoregulatory capacity, potentially resulting women with obesity to enter pregnancy with a pre-existing immunoregulatory deficit that increases GDM susceptibility (94). Women with obesity and GDM exhibit upregulated Th1-associated cytokines and transcription factors, including IL-2, IFN-γ, and T-bet (95). This pattern is consistent with a pro-inflammatory immune phenotype that may contribute to impaired insulin signalling and endothelial dysfunction (95). Collectively, these innate and adaptive immune pathway alterations illustrate how obesity-driven inflammation disrupts immunometabolic homeostasis and may contribute to GDM susceptibility.

## Placental immunology and inflammation in obesity-related GDM

Once GDM has developed, persistent maternal hyperglycaemia and systemic inflammation further alter placental immune function, leading to increased inflammatory signalling and disruption of placental homeostasis (96, 97). These secondary changes may contribute to adverse foetal programming and increase the risk of long-term metabolic disease in the offspring (98, 99). Although placental dysfunction may also participate in disease progression, current evidence suggests that many placental immune alterations are better interpreted as amplifying, rather than initiating, mechanisms in obesity-associated GDM. This is consistent with previous work proposing that maternal obesity and systemic inflammation initiate immunometabolic dysfunction, whereas placental inflammatory pathways primarily adapt to and reinforce disease progression once hyperglycemia has developed (12).

Hofabuer Cells (HBCs) exhibit considerable phenotypic and functional plasticity, and literature regarding their phenotype and activation status in GDM remains elusive. The reported differences across studies may reflect biological and methodological heterogeneity rather than real contradictions. Therefore, the phenotypic and functional profiles of HBCs reported in GDM may be potnentially influenced by gestational age, maternal metabolic status, obesity severity, placental tissue samples and varying experimental approaches. Nonetheless, various studies suggest that HBCs retain their anti-inflammatory characteristics despite maternal hyperglycaemia (55, 96, 97, 100–102), others have reported increased M1 macrophage polarisation in GDM placentas, supporting a shift towards a more pro-inflammatory phenotype (55, 103). Bayramoğlu and colleagues identified increased expression of CD4, CD8, CD68, CD80 and CD86 in GDM placentas, providing further evidence of enhanced immune-cell activation at the maternal-foetal interface (104). Maternal hyperglycaemia and metabolic stress may further promote placental inflammation through activation of the NLRP3 inflammasome and increased expression of TNF-α, IL-6 and IL-17A, accompanied by enrichment of Th1 and Th17 lymphocyte subsets (74, 89, 105). Collectively, these changes are consistent with enhanced placental immune activation at the maternal-foetal interface and may contribute to placental dysfunction. Although GDM appears to be associated with enhanced placental immune activation, evidence for a specific shift in HBC polarisation towards an M1 or M2 phenotype in GDM remains inconclusive.

Evidence regarding HBC abundance is similarly inconsistent, with studies reporting both increases and decreases in HBC density in GDM placentas (102, 106). Increased HBC abundance may contribute to a more inflammatory placental environment by enhancing local cytokine production, irrespective of macrophage phenotype (97). Importantly, changes in HBC abundance should not be interpreted as evidence of altered phenotype or function, as quantitative differences may occur independently of functional polarisation.

Discrepancies in HBC polarisation and abundance between studies may reflect differences in disease severity, gestational age, tissue sampling and markers and classification systems used to define macrophage subsets. This variety highlights the need for standardised phenotypic and functional characterisation of HBCs in GDM.

GDM is also associated with morphological alterations in the placenta, including reduced vascularisation and impaired villous maturation. These changes may compromise syncytiotrophoblast function, increase cellular stress, and promote the release of pro-inflammatory mediators (97, 106–108). In addition, GDM has been associated with altered placental nutrient transport, although available evidence remains inconsistent. Some studies report increased expression of glucose transporters, particularly GLUT1 (109, 110), whereas others observe no significant changes in transplacental glucose transfer (111). Similarly, dysregulated lipid transport has been linked to altered AMP-activated protein kinase (AMPK) signalling and increased TNF-α expression at the fetoplacental interface, although findings regarding fatty acid transport and lipid metabolism remain conflicting (109, 112). Together, these structural and immunological alterations may impair placental function and contribute to adverse foetal development. Consequently, maternal obesity and GDM are associated with an increased risk of long-term offspring metabolic complications, highlighting the placenta as a potential mediator of immunometabolic dysfunction and intergenerational metabolic risk.

Taken together, these findings suggest that the placenta functions as both a target and an amplifier of obesity-associated immune dysregulation. Rather than being the primary initiator of GDM, placental immune alterations may primarily reinforce maternal metabolic dysfunction and contribute to the downstream consequences of established disease. However, placental alterations may also contribute to early metabolic deterioration at the maternal-foetal interface. Thus, the directionality and temporal relationship between placental immune alterations and maternal metabolic dysfunction remain uncertain.

## Conclusions

Pre-pregnancy obesity represents a major and modifiable risk factor for GDM, with immune dysregulation increasingly recognised as an important component of its pathophysiology. The convergence of chronic inflammation, altered immune cell function, and disrupted cytokine and adipokine networks contributes to heightened insulin resistance, β-cell dysfunction, and IL-6impaired placental adaptation. These findings underscore the importance of considering GDM not solely as a metabolic disorder but as an immunometabolic condition shaped by pre-existing inflammatory states. Despite advances in understanding, significant gaps remain in delineating the temporal dynamics and causal pathways linking immune alterations to metabolic dysfunction in pregnancy. Future research should prioritise longitudinal and mechanistic studies, particularly in diverse populations, to better define the immunometabolic pathways linking obesity to GDM, and establish their chronology and causality. Importantly, understanding immune pathways may identify novel opportunities for early risk stratification, intervention, and prevention of GDM, ultimately improving maternal and offspring health outcomes in the context of the global obesity epidemic.
